# A single nucleotide substitution in *TaHKT1*;*5‐D* controls shoot Na^+^ accumulation in bread wheat

**DOI:** 10.1111/pce.13841

**Published:** 2020-07-22

**Authors:** Chana Borjigin, Rhiannon K. Schilling, Jayakumar Bose, Maria Hrmova, Jiaen Qiu, Stefanie Wege, Apriadi Situmorang, Caitlin Byrt, Chris Brien, Bettina Berger, Matthew Gilliham, Allison S. Pearson, Stuart J. Roy

**Affiliations:** ^1^ Australian Centre for Plant Functional Genomics, The University of Adelaide Glen Osmond South Australia Australia; ^2^ School of Agriculture, Food and Wine, The University of Adelaide Glen Osmond South Australia Australia; ^3^ ARC Centre of Excellence in Plant Energy Biology The University of Adelaide Glen Osmond South Australia Australia; ^4^ School of Life Sciences, Huaiyin Normal University Huai'an China; ^5^ Division of Plant Sciences Research School of Biology, Australian National University Acton Australian Capital Territory Australia; ^6^ Australian Plant Phenomics Facility The Plant Accelerator, The University of Adelaide Glen Osmond South Australia Australia; ^7^ ARC Industrial Transformation Research Hub for Wheat in a Hot Dry Climate, The University of Adelaide Glen Osmond South Australia Australia

**Keywords:** Na^+^ transport, plant growth, salt tolerance, sodium, xylem sap Na^+^

## Abstract

Improving salinity tolerance in the most widely cultivated cereal, bread wheat (*Triticum aestivum* L.), is essential to increase grain yields on saline agricultural lands. A Portuguese landrace, Mocho de Espiga Branca accumulates up to sixfold greater leaf and sheath sodium (Na^+^) than two Australian cultivars, Gladius and Scout, under salt stress in hydroponics. Despite high leaf and sheath Na^+^ concentrations, Mocho de Espiga Branca maintained similar salinity tolerance compared to Gladius and Scout. A naturally occurring single nucleotide substitution was identified in the gene encoding a major Na^+^ transporter TaHKT1;5‐D in Mocho de Espiga Branca, which resulted in a L190P amino acid residue variation. This variant prevents Mocho de Espiga Branca from retrieving Na^+^ from the root xylem leading to a high shoot Na^+^ concentration. The identification of the tissue‐tolerant Mocho de Espiga Branca will accelerate the development of more elite salt‐tolerant bread wheat cultivars.

## Abstract 2

A bread wheat (*Triticum aestivum* L.) landrace Mocho de Espiga Branca maintained similar salinity tolerance compared to commercial cultivars despite accumulating high leaf sodium. An impaired sodium transporter *TaHKT1;5‐D* was shown to be responsible for the increased shoot sodium in this landrace.

## 1 INTRODUCTION

Globally, 45 million ha of irrigated and 32 million ha of dryland agricultural land is affected by salinity (FAO, 2019a). It is estimated that up to 1.2 million ha of land is lost to salinization each year (FAO, 2019a). To feed the rapidly growing human population, global food production must increase more than 70% by 2050, equating to an average increase of 44 million metric tons per year (Tester & Langridge, 2010). Bread wheat (*Triticum aestivum*) is the most widely cultivated cereal crop, in terms of area and provides one fifth of the total calories consumed worldwide (FAO, 2019b). Improving the salinity tolerance of bread wheat to maximize yields on saline agricultural land is required.

Salinity affects plants in two distinct stages. Shoot ion‐independent stress (osmotic stress) arises immediately after plants are exposed to salt, inducing rapid physiological responses, such as stomatal closure and slower cell expansion, resulting in reduced plant growth (Munns & Tester, 2008). Shoot ion‐dependent stress (ionic stress) has a slower onset and occurs when salt, particularly Na^+^ and Cl^−^, accumulate to high concentrations in the shoot (Munns & Tester, 2008). In this phase, reduced plant growth becomes more evident and premature leaf senescence occurs due to the toxicity of salt on cell metabolism (Munns & Tester, 2008).

Plants have three main mechanisms for tolerating salinity: ionic tissue tolerance by compartmentalizing excessive Na^+^ or Cl^−^ in vacuoles to avoid accumulation to toxic concentrations in the cytoplasm, the generation of compatible solutes and the detoxification of reactive oxygen species (Flowers, Munns, & Colmer, 2015; Munns, James, Gilliham, Flowers, & Colmer, 2016; Munns & Tester, 2008); the exclusion of Na^+^ from the shoot by retrieving Na^+^ from the xylem into the root or through efflux of Na^+^ into the soil to maintain a low shoot Na^+^ concentration (Byrt et al., 2014; Møller et al., 2009; Munns & Tester, 2008); and osmotic stress tolerance leading to continued growth, albeit at a lower rate than non‐salt exposed plants (Munns & Tester, 2008). The mechanisms that lead to osmotic tolerance are less well defined but involves long distance signalling (Munns & Tester, 2008; Steinhorst & Kudla, 2019; Suzuki, Koussevitzky, Mittler, & Miller, 2012).

The high‐affinity potassium transporter 1;5 (HKT1;5) is known to be responsible for retrieving Na^+^ from the transpiration stream in the root and preventing Na^+^ from accumulating to high concentrations in the shoot (Hamamoto et al., 2015). Plant HKT proteins belong to the high‐affinity K^+^/Na^+^ transporters Ktr/TrK/HKT superfamily and are divided into two groups based on a serine/glycine substitution in the first loop of the proteins (Platten et al., 2006). Members of the HKT1 group with a serine residue, typically transport Na^+^, while the HKT2 group with a glycine side chain generally transports both Na^+^ and K^+^ (Platten et al., 2006). In Arabidopsis (*Arabidopsis thaliana*) overexpression of *AtHKT1*;*1* in root stelar cells reduced shoot Na^+^ accumulation by up to 64% compared to null segregants (Møller et al., 2009). In bread wheat, the *AtHKT1*;*1* orthologue, *TaHKT1*;*5‐D*, is also expressed in root stelar cells and reduces shoot Na^+^ accumulation under salinity (Byrt et al., 2014). Durum wheat (*Triticum turgidum* L.), which lacks the D‐genome accumulates high concentrations of Na^+^ in the leaf (Munns, Rebetzke, Husain, James, & Hare, 2003). When *TmHKT1;5‐A* (*Nax2* locus) from a wheat relative *Triticum monococcum* L. was introduced into a commercial durum wheat, a reduction in leaf Na^+^ concentration (James, Davenport, & Munns, 2006) and a 25% improvement in grain yield in the field were observed (Munns et al., 2012).

Breeding of salt‐tolerant bread wheat cultivars has focused on selecting genotypes with improved shoot Na^+^ exclusion (Ashraf & O'leary, 1996; Poustini & Siosemardeh, 2004). However, selection based on shoot Na^+^ exclusion is not always correlated with increased salinity tolerance in bread wheat (Genc et al., 2013; Genc et al., 2019). Barley (*Hordeum vulgare* L.) is one of the most salt‐tolerant cereal crops and has much higher leaf Na^+^ concentrations than bread wheat, yet it is able to maintain shoot growth in saline soils (Munns & Tester, 2008; Tilbrook et al., 2017). The identification of a bread wheat line that accumulates high shoot Na^+^ concentrations whilst maintaining salinity tolerance, similar to barley, may accelerate the development of more salt‐tolerant bread wheat cultivars.

Here, we identified a Portuguese bread wheat landrace Mocho de Espiga Branca, which accumulated significantly higher leaf Na^+^ concentrations while maintaining similar salinity tolerance as current commercial elite bread wheat cultivars. A naturally occurring single nucleotide polymorphism (SNP) in *TaHKT1*;*5‐D* prevents Mocho de Espiga Branca from retrieving Na^+^ from the root xylem, which results in a greater flux of Na^+^ to the shoot and higher accumulation of Na^+^ in leaf tissues.

## 2 MATERIALS AND METHODS

### 2.1 Plant materials and growth condition

Two Australian commercial bread wheat cultivars Gladius and Scout and a set of 73 bread wheat diversity lines consisting of advanced cultivars, landraces and synthetic hexaploids were initially screened for salinity tolerance in this study (Garcia et al., 2019). In the subsequent physiological characterizations, one of the 73 bread wheat diversity lines, a Portuguese landrace Mocho de Espiga Branca, and the commercial cultivars Gladius and Scout were used. Plants in all the glasshouse experiments were grown under natural light with a daytime temperature at 22°C and 15°C at night.

### 2.2 Screening of bread wheat diversity set in a pot trial

A total of 75 lines (Gladius and Scout together with the 73 diversity lines) were screened for salinity tolerance in a fully automated conveyor system within a temperature controlled Smarthouse at the Australian Plant Phenomics Facility (The Plant Accelerator, University of Adelaide, Australia; Latitude: −34.971366°, Longitude: 138.639758°) between August and September of 2014. Uniform sized seeds of each line were placed into 50 ml polypropylene tubes and imbibed in reverse osmosis (RO) water at room temperature for 4 hr, tubes were drained and placed in the dark at 4°C for 3 days before sowing. Three seeds from each line were sown in a free‐draining plastic pot (145 mm diameter × 190 mm height) filled with 2.5 kg of a soil mixture (50% (v/v) University of California mix, 35% (v/v) peat mix and 15% (v/v) clay loam). The pots were arranged according to a split‐plot experimental design with two consecutive pots forming a main plot. The lines were allocated to the main plots and were unequally replicated with Gladius and Scout replicated 12 times, 21 randomly selected lines of the 73 diversity lines were replicated four times and the remaining 52 lines were replicated three times. The subplot design randomized the two treatment levels (0 and 100 mM NaCl) in each main plot. The main plot design was a blocked row‐and‐column design generated using DiGGer (Coombes, 2009) and the subplot randomization was generated using dae (Brien, 2014), packages for the R statistical computing environment (R Core Team, 2014). At the emergence of the second leaf, plants were thinned to one uniformly sized plant per pot. At the emergence of the third leaf, pots were loaded onto an individual cart in the Smarthouse, where they were weighed daily and automatically watered to maintain the soil water content in each pot at 17% (w/w). At the emergence of the fourth leaf, 213 ml of either 0 or 170 mM NaCl solution was applied to a saucer below each pot. Plants were not watered again until the soil water content reduced below 17% (w/w), and then each pot was watered again daily to maintain a final treatment concentration of 0 or 100 mM NaCl in the respective pot.

The shoot area of each plant was non‐destructively imaged using a LemnaTec 3D Scanalyzer system (LemnaTec GmbH) for a total of 15 days (4 days before and 11 days after the NaCl treatment). Three red–green–blue (RGB) images (one top view and two side view images with a 90° angle) were recorded daily for each plant to calculate the projected shoot area (PSA) in pixels. Eleven days after the NaCl treatment, the fully expanded fourth leaf blade of each plant was collected, weighed and dried in an oven at 60°C for 2 days before the dry weight was recorded. The dried fourth leaf blade was subsequently used for measuring Na^+^, K^+^ and Cl^−^ concentrations.

### 2.3 Determining leaf blade, leaf sheath and root Na^+^ concentration in hydroponics

To measure the Na^+^, K^+^ and Cl^−^ concentrations in the fourth leaf blade and sheath and roots, Mocho de Espiga Branca, Gladius and Scout were grown using a fully supported hydroponics system under three concentrations of NaCl (0, 150 and 200 mM) in a controlled glasshouse at The Plant Accelerator between June and August 2017. The hydroponic system was equipped with a trolley fitted with two growth trays each containing 42 tubes filled with polycarbonate pellets and a tank containing 80 L of a modified Hoagland solution (mM): NH_4_NO_3_ (0.2); KNO_3_ (5.0); Ca(NO_3_)_2_·4H_2_O (2.0); MgSO_4_·7H_2_O (2.0); KH_2_PO_4_ (0.1); Na_2_Si_3_O_7_ (0.5); NaFe(III)EDTA (0.05); MnCl_2_·4H_2_O (0.005); ZnSO_4_·7H_2_O (0.01); CuSO_4_·5H_2_O (0.0005) and Na_2_MoO_3_·2H_2_O (0.0001). Uniform sized seeds from each genotype were surface sterilized using ultraviolet (UV) light for 2 min and germinated in Petri dishes on moist filter paper for 4 days at room temperature before transplanting. Fourteen replicates from each cultivar were grown in each treatment trolley. The NaCl treatment was applied at the emergence of the fourth leaf by adding 116.88 g of NaCl twice daily for a 25 mM NaCl increment until a final concentration of either 150 mM or 200 mM was reached, and no NaCl was added to the 0 mM NaCl treatment. The 3.8 g of supplemental CaCl_2_·2H_2_O was added into the 150 and 200 mM NaCl treatment tanks at each 25 mM NaCl increment in order to maintain constant Ca^2+^ activity in all three treatments. The plants were irrigated by the nutrient solution in a 20 min flood and drain cycle and the complete nutrient solution was replaced every 7 days. The pH of the solution was maintained between 6.5 and 7.0 throughout the experiment using a 3.2% (v/v) HCl solution and a portable waterproof specific Ion–pH–mV–Temperature metre (Modal WP‐90; TPS Pty Ltd, Australia). After 21 days of NaCl treatment, the fully expanded fourth leaf blade and sheath and the remaining shoots were sampled separately and weighed. Plant roots were weighed after sampling and approximately 5 cm of the root tip was used for RNA extraction. The roots from the 150 mM and 200 mM treatments were rinsed in 10 mM CaSO_4_ solution and blotted on paper towel to remove traces of NaCl before sampling. The weighed fourth leaf blade, fourth leaf sheath, shoots and roots were dried in an oven at 60°C for 2 days to record the dry weight. The dried fourth leaf blade and sheath and root tissue were used for the subsequent Na^+^, K^+^ and Cl^−^ concentration analysis.

### 2.4 Na^+^, K^+^ and Cl^−^ concentration analysis

The harvested dried fourth leaf blade and root samples were digested in 10 ml of 1% (v/v) HNO_3_ (v/v), and the fourth leaf sheath was digested in 5 ml of 1% (v/v) HNO_3_ (v/v) at 85°C for 4 hr in a SC154 HotBlock (Environmental Express Inc., South Carolina, USAS). Na^+^ and K^+^ concentrations were measured using a flame photometer (Model 420; Sherwood Scientific Ltd., Cambridge, UK), and Cl^−^ concentration was measured using a chloride analyzer (Model 926; Sherwood Scientific Ltd., Cambridge, UK).

### 2.5 RNA extraction and cDNA synthesis

The harvested 5 cm root tips from the hydroponics experiment were snap frozen in liquid nitrogen and stored at −80°C until RNA extraction. The root tissue was ground to a fine powder using a 2010 Geno/Grinder (SPEX SamplePrep) at 1,000 *g* for 30 s, and total RNA was extracted from the tissue powder using a Direct‐Zol RNA MiniPrep kit with DNase I treatment (Zymo Research) according to the manufacturer's instruction. Final elution was performed with 40 μl DNA/RNAase‐Free water supplied and the eluted RNA was subsequently quantified using a ND‐1000 Spectrophotometer (NanoDrop Technologies) and quantified on a 1% (w/v) agarose gel (Bioline) by electrophoresis. cDNA synthesis was performed on 500 ng of RNA using High Capacity cDNA Reverse Transcription Kit (Thermo Fisher Scientific) according to the manufacturer's instruction in a 20 μl reaction and stored at −20°C until use.

### 2.6 *TaHKT1*;*5‐D* coding sequence amplification and sequencing

The entire coding sequence of the *TaHKT1*;*5‐D* gene from Mocho de Espiga Branca, Gladius and Scout was amplified using Phusion High‐Fidelity DNA polymerase (New England Biolabs, Inc., Beverly, MA, USA) following the manufacturer's instruction. The primers used for PCR amplification were: forward primer *cTaHKT1*;*5‐D_FP_1* (5’‐ATGGGTTCTTTGCATGTCTCCT‐3′) and reverse primer *cTaHKT1*;*5‐D_RP_1551* (5′‐TTATACTATCCTCCATGCCTCGC‐3′) (Table S3). PCR was conducted on a T100 Thermal Cycler (Bio‐Rad, Hercules, CA, USA) using the following conditions: initial denaturation at 98°C for 30 s, 35 cycles of 98°C for 30 s, 64°C for 30 s, 72°C for 1 min, final extension at 72°C for 10 min and held at 4°C. The PCR product was visualized on a 1% (w/v) agarose gel (Bioline) by electrophoresis at 90 V for 1 hr and the 1.5 kb target band was collected for purification using NucleoSpin Gel and PCR Clean‐up kit (Macherey‐Nagel) according to the manufacturer's instruction prior to sequencing. Three replicates from each of Mocho de Espiga Branca, Gladius and Scout *TaHKT1*;*5‐D* coding sequence were tested with primers listed in Table S3 for Sanger sequencing carried out at the Australian Genome Research Facility (AGRF, South Australia).

### 2.7 *TaHKT1*;*5‐D* gene expression in roots

To determine *TaHKT1*;*5‐D* expression in Mocho de Espiga Branca, Gladius and Scout, a PCR was conducted on the synthesized cDNA in a 25 μl reaction consisting of 1 μl cDNA, 0.5 μl each of 10 μM forward primer 5′‐CGACCAGAAAAGGATAACAAGCAT‐3′ and reverse primer 5′‐AGCCAGCTTCCCTTGCCAA‐3′, 5 μl *Taq* 5× Master Mix (New England Biolabs) and 18 μl Milli‐Q water (18.2 MΩ cm). The final PCR products were visualized on a 1% (w/v) agarose gel (Bioline) by electrophoresis at 90 V for 1 hr. The targeted *TaHKT1*;*5‐D* product was 283 bp. The *TaGAP* gene (230 bp) was used as a positive control and a Milli‐Q water sample was included as a negative control.

To quantify *TaHKT1*;*5‐D* expression in the root tissue of Mocho de Espiga Branca, Gladius and Scout, Real‐time PCR was performed on five replicates from each of the cultivars using an Applied Biosystems QuantStudio 6 Flex (Life Technologies, Rockville, MD, USA). The reaction was performed in a 10 μl reaction consisting of 2 μl 1:20 diluted synthesized cDNA, 0.5 μl each of 10 μM forward and reverse primers stated above, 5 μl KAPA SYBR FAST 2× Master Mix (Sigma‐Aldrich, St Louis, MO, USA) and 2 μL Milli‐Q water. The 283 bp final product from each cultivar was confirmed by Sanger sequencing carried out at AGRF (South Australia).

### 2.8 DNA extraction and quantification

Genomic DNA (gDNA) extraction of Mocho de Espiga Branca, Gladius, Scout and 70 bread wheat diversity lines was performed using a phenol/chloroform/iso‐amyl alcohol extraction method as described elsewhere with modifications (Rogowsky, Guidet, Langridge, Shepherd, & Koebner, 1991). Briefly, the leaf tissue was frozen in a 10 ml tube at −80°C and ground to a fine powder using a 2,600 Cryo‐Station (SPEX SamplePrep) and a 2010 Geno/Grinder (SPEX SamplePrep) at 1,000 *g* for 30 s. About 2 ml of gDNA extraction buffer [1% (w/v) sarkosyl, 100 mM Tris–HCl, 100 mM NaCl, 10 mM EDTA, 2% (w/v) insoluble polyvinyl‐polypyrrolidone] was added to the ground tissue, vortexed followed by the addition of 2 ml phenol/chloroform/iso‐amyl alcohol (25:24:1). The sample was placed on ice for 20 min and vortexed thoroughly in every 5 min before centrifuging at 3,630*g* for 15 min. The supernatant was transferred into a labelled BD Vacutainer SST II Advance tube (Becton, Dickinson and Company, NJ, USA) and 2 ml of phenol/chloroform/iso‐amyl alcohol (25:24:1) was added. The sample was vortexed and centrifuged as above, and the supernatant was collected into a new 10 ml tube. The gDNA was precipitated using 2 ml of 100% (v/v) iso‐propanol and 200 μl of 3 M sodium acetate (pH 4.8) and washed using 1 ml of 70% (v/v) ethanol before re‐suspending overnight in 80 μl of R40 at 4°C. The re‐suspended gDNA was quantified using a ND‐1000 Spectrophotometer (NanoDrop Technologies).

### 2.9 Cleaved amplified polymorphic sequence (CAPS) assay and genotyping

A CAPS marker tsl2SALTY‐4D was designed to confirm the allele effect of the SNP (T/C) identified in Mocho de Espiga Branca *TaHKT1*;*5‐D* for high leaf Na^+^ concentration in comparison to Gladius and Scout. The extracted DNA from Mocho de Espiga Branca, Gladius, Scout and 68 bread wheat diversity lines were used for a PCR to amplify a 945 bp DNA fragment containing SNP. The PCR analysis was conducted in a 10 μl reaction consisting of 1 μg of DNA, 0.24 μl each of 10 μM forward primer 5′**‐**ATGGGTTCTTTGCATGTCTCCT**‐**3′ and reverse primer 5′**‐**CGCTAGCACGAACGCCG**‐**3′, 2 μl of *Taq* 5× Master Mix (New England Biolabs) and Milli‐Q water. The reaction was performed on a T100 Thermal Cycler (Bio‐Rad) using the following conditions: initial denaturation at 95°C for 4 min, 35 cycles of 95°C for 30 s, 56°C for 30 s, 68°C for 1 min, final extension at 68°C for 5 min and held at 12°C. The PCR amplification was followed by digestion using the restriction enzyme *FauI*. It was conducted in a 10 μl reaction consisting of 1 μg of the PCR products, 0.4 μl of *FauI* enzyme (New England Biolabs), 1 μl of 10× CutSmart Buffer (New England Biolabs) and Milli‐Q water. The digestion was performed on a T100 Thermal Cycler (Bio‐Rad) for 1 hr at 55°C followed by 20 min of inactivation at 65°C and held at 12°C. The digested product was visualized on a 2% (w/v) agarose gel (Bioline) by electrophoresis at 90 V for 90 min and the genotype of each line at the SNP position was confirmed according to the product bands on the gel. Lines which had the C:C genotype had two fragments present at 573 and 372 bp, whilst lines carrying the T:T genotype had a single fragment present.

### 2.10 Construction of 3D molecular models of *TaHKT1*;*5‐D* and *TaHKT1*;*5‐D L190P*

The most suitable template for wheat HKT1;5 transporter proteins was the *B. subtilis* KtrB K^+^ transporter (Protein Data Bank accession 4J7C, chain I) (Vieira‐Pires, Szollosi, & Morais‐Cabral, 2013) as previously identified (Xu et al., 2018). The K^+^ ion in KtrB was substituted by Na^+^ during modelling of TaHKT1;5 proteins. Three‐dimensional models of TaHKT1;5‐D and TaHKT1;5‐D L190P in complex with Na^+^ were generated with Modeller 9v19 (Sali & Blundell, 1993) as described previously (Cotsaftis, Plett, Shirley, Tester, & Hrmova, 2012; Waters, Gilliham, & Hrmova, 2013) incorporating Na^+^ ionic radii (Xu et al., 2018) taken from the CHARMM force field (Brooks et al., 2009), on the Linux station running the Ubuntu 12.04 operating system. Best scoring models (from an ensemble of 50) were selected based on the combination of Modeller Objective (Shen & Sali, 2006) and Discrete Optimized Protein Energy (Eswar, Eramian, Webb, Shen, & Sali, 2008) functions, PROCHECK (Laskowski, Macarthur, Moss, & Thornton, 1993), ProSa 2003 (Sippl, 1993) and FoldX (Schymkowitz et al., 2005). Structural images were generated in the PyMOL Molecular Graphics System V1.8.2.0 (Schrődinger LLC, Portland, OR, USA). Calculations of angles between selected α‐helices in HKT1;5 models were executed in Chimera (Pettersen et al., 2004) and evaluations of differences (ΔΔG = ΔGmut‐ΔGwt) of Gibbs free energies was performed with FoldX (Schymkowitz et al., 2005). Sequence conservation patterns were analysed with ConSurf (Celniker et al., 2013; Landau et al., 2005) based on 3D models of TaHKT1;5‐D using 370 sequences at the sequence identities of 30% and higher (specifications: HMMMER homologue search algorithm, UNIREF‐90 Protein database with the E‐value cut‐off of 1 × 10^−4^, Bayesian Model of substitution for proteins.

### 2.11 Characterization of *TaHKT1*;*5‐D* from Mocho de Espiga Branca and Gladius in *X. laevis* oocytes

Na^+^ transport properties of TaHKT1;5‐D from Mocho de Espiga Branca and Gladius were characterized in *X. laevis* oocytes using two‐electrode voltage clamping (TEVC) as previously described (Byrt et al., 2014; Munns et al., 2012). pGEMHE‐DEST containing *TaHKT1*;*5‐D* was linearized using sbfI‐HF (New England Biolabs) before synthesizing cRNA using the mMESSAGE mMACHINE T7 Kit (Ambion, Austin, TX, USA) following manufacturer's instructions. The 46 nl/23 ng of the cRNA from Mocho de Espiga Branca or Gladius, or equal volumes of RNA‐free water (H_2_O control) were injected into oocytes. Injected oocytes were incubated for 48 hr and TEVC was carried out as described (Munns et al., 2012). Membrane currents were recorded in the HMg solution (6 mM MgCl_2_, 1.8 mM CaCl_2_, 10 mM MES, and pH 6.5 adjusted with a Tris base) ± Na^+^ glutamate. The osmolality of the solution was adjusted to 240–260 mOsmol Kg^−1^ using mannitol and a micro‐osmometer (Model 210; Fiske Associates Inc, USA).

### 2.12 Subcellular localization of *TaHKT1*;*5‐D*

Transient expression of fluorescent fusion proteins was performed as previously described (Henderson et al., 2015). In brief, *TaHKT1*;*5‐D* coding sequences of Mocho de Espiga Branca and Gladius were recombined into pMDC43 (Curtis & Grossniklaus, 2003) to generate N‐terminally green fluorescent protein (GFP) tagged proteins. The red fluorescent protein (RFP) tagged plasma membrane marker nCBL1‐RFP that was used for co‐localization (Batistic, Sorek, Schultke, Yalovsky, & Kudla, 2008). All constructs were transformed into *Agrobacterium tumefaciens* strain Agl‐1 and agro‐infiltration was performed on fully expanded leaves of 4–6 week‐old tobacco (*Nicotiana benthamiana*) plants (Henderson et al., 2015). After 2–3 days, leaf sections were imaged using a Nikon A1R Confocal Laser‐Scanning Microscope equipped with a 63× water objective lens and NIS‐Elements C software (Nikon Corporation). Excitation/emission conditions were GFP (488 nm/500–550 nm) and RFP (561 nm/570–620 nm).

### 2.13 Xylem sap Na^+^ and K^+^ concentration analysis

Xylem sap was collected from hydroponically grown plants of Mocho de Espiga Branca and Gladius at 0 and 150 mM NaCl after 21 days from the start of the NaCl treatment. The shoot was cut off at the base of the plant and inserted into a Scholander‐type pressure chamber (Model 1005; PMS Instrument Company) to extrude the xylem sap by slowly filling the chamber with compressed air. The sap was immediately collected into a clean, pre‐weighed 1.5 ml tube. From the 0 mM NaCl treatment, xylem sap of seven plants from each cultivar was collected, and xylem sap of eight Mocho de Espiga Branca and seven Gladius plants was collected from the 150 mM NaCl treatment. Tubes containing the xylem sap was weighed for each plant and the samples were stored at 4°C until Na^+^ and K^+^ concentrations were measured using a flame photometer (Model 420; Sherwood Scientific Ltd., Cambridge, UK).

### 2.14 Net and total Na^+^, K^+^ and H^+^ flux measurements using microelectrode ion flux estimation (MIFE) technique

To investigate whether there were differences in Na^+^, K^+^ and H^+^ transport in the plant roots between Mocho de Espiga Branca and Gladius, net fluxes of Na^+^, K^+^ and H^+^ were measured at root maturation and elongation zones using the non‐invasive MIFE technique (University of Tasmania, Hobart, Australia) (Bose et al., 2015; Newman, 2001).

Root Na^+^ retrieval measurements were performed at the elongation zone (approximately 600 μm from the root cap). Uniform sized sterilized seeds from Mocho de Espiga Branca and Gladius were germinated on moist filter paper in Petri dishes covered with aluminium foil, at 4°C overnight and then placed at room temperature in the dark for 3 days before transplanting. Twelve seedlings from each cultivar were transplanted into a hydroponic tank containing 10 L modified Hoagland solution as previously described. NaCl was added into the solution at an increment of 25 mM (14.16 g) twice daily for 2 days to achieve a final concentration of 100 mM NaCl. The pH of the solution was maintained between 6.5 and 7.0 throughout the experiment using 3.2% (v/v) of the HCl solution and a portable waterproof specific Ion–pH–mV–Temperature metre (Modal WP‐90; TPS Pty Ltd, Australia). Two days after being exposed to 100 mM NaCl, the entire roots of Mocho de Espiga Branca (*n* = 9) and Gladius (*n* = 8) were first preconditioned in BSM solution containing 100 mM NaCl (0.2 mM KCl + 0.1 mM CaCl_2_·2H_2_O + 100 mM NaCl) in a Petri dish for 30 min and then the primary root was immobilized on a 10 ml perspex measuring chamber containing 7 ml of the same BSM medium. The steady‐state fluxes were measured for 5 min in the initial BSM solution before changing the bathing solution to 7 ml of the new BSM medium solution containing 0.6 mM NaCl (0.2 mM KCl + 0.1 mM CaCl_2_·2H_2_O + 0.6 mM NaCl). The resulting fluxes were measured for 25 min and the integral of each replication was added to derive the cumulative total fluxes. The osmolality of the two BSM solutions and the Hoagland solution were maintained between 208 and 222 mOsmol kg^−1^ using mannitol and a vapour pressure osmometer (Model 5500; Wescor, Inc., USA).

Root Na^+^ uptake measurements were performed at the maturation zone (beyond 2.5 cm from root tip), uniform sized sterilized seeds were germinated as described above, placed at 4°C overnight and placed at room temperature in the dark for 3 days before measurement. The primary root of Mocho de Espiga Branca (*n* = 12) and Gladius (*n* = 11) seedlings was immobilized on a 10 ml perspex measuring chamber containing 6 ml of the BSM solution (0.2 mM KCl + 0.1 mM CaCl_2_·2H_2_O + 0.6 mM NaCl) and preconditioned for 20 min before recording steady‐state Na^+^, K^+^ and H^+^ fluxes for 5 min then 100 mM NaCl was added and the resulting fluxes were recorded for 25 min.

### 2.15 Statistical analysis

Prism 7 for Windows (version 7.02; GraphPad Software, Inc.) was used to generate graphs. GenStat 15th edition for Microsoft Windows (version 15.3.09425; VSN International Ltd, UK) was used to perform an Analysis of Variance (ANOVA) and Tukey's multiple comparison test was used to determine which means were significantly different at a probability level of *p* ≤ .05.

## 3 RESULTS

### 3.1 Mocho de Espiga Branca has high leaf and sheath Na^+^ concentrations

**FIGURE 1:**
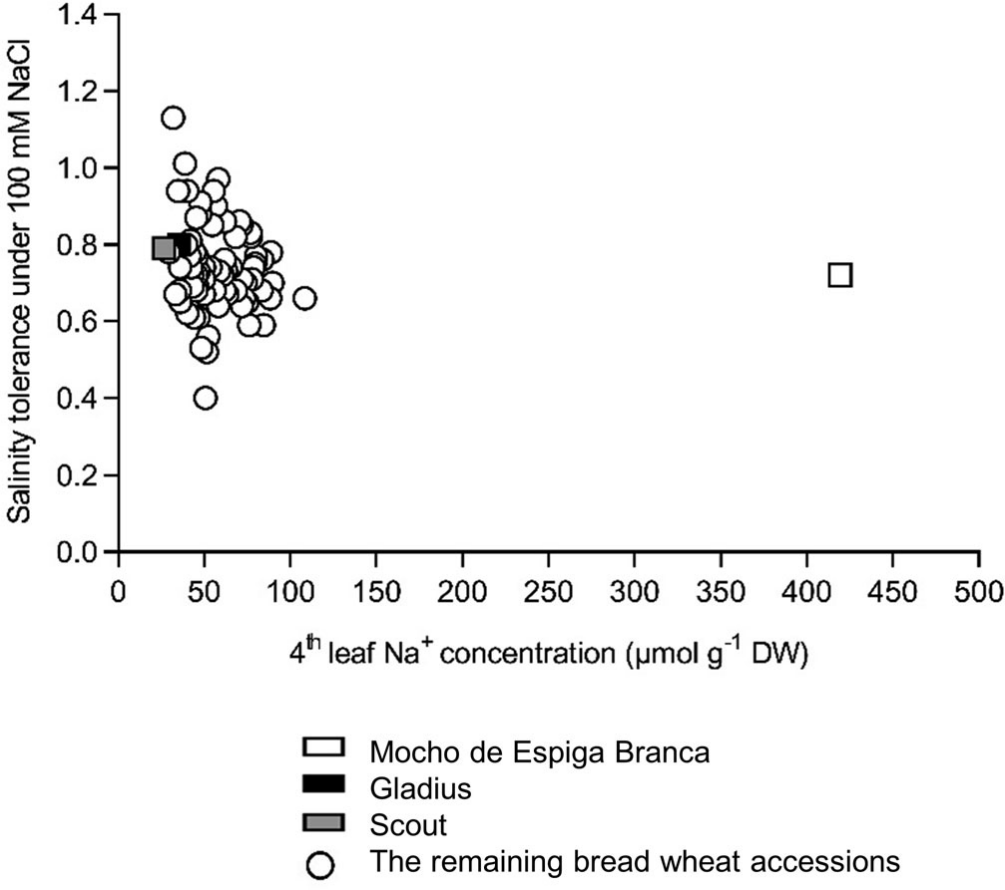
Salinity tolerance and fourth leaf Na^+^ concentration of Mocho de Espiga Branca relative to 72 bread wheat diversity lines and two Australian cultivars Gladius and Scout in soil with 100 mM NaCl. The fourth leaf Na^+^ concentration is determined 11 days after treatment with 100 mM NaCl. Salinity tolerance is defined as projected shoot area (PSA) under 100 mM NaCl relative to 0 mM NaCl determined from the final day of imaging. Data presented as means (*n* = 3–4 except for Gladius and Scout, where *n* = 12). The standard error of the mean (SEM) for the fourth leaf Na^+^ concentration is presented in Table S1

Screening of 75 bread wheat accessions (including the Australian cultivars Gladius and Scout) in soil for salinity tolerance under 100 mM NaCl, identified a Portuguese landrace, Mocho de Espiga Branca, which had a similar salinity tolerance but accumulated a sixfold higher Na^+^ concentration in the fourth leaf compared to the other 74 lines (Figure 1 and Table S1). The fourth leaf K^+^ and Cl^−^ concentrations of the 75 wheat lines were comparable under 100 mM NaCl (Table S1).

**FIGURE 2:**
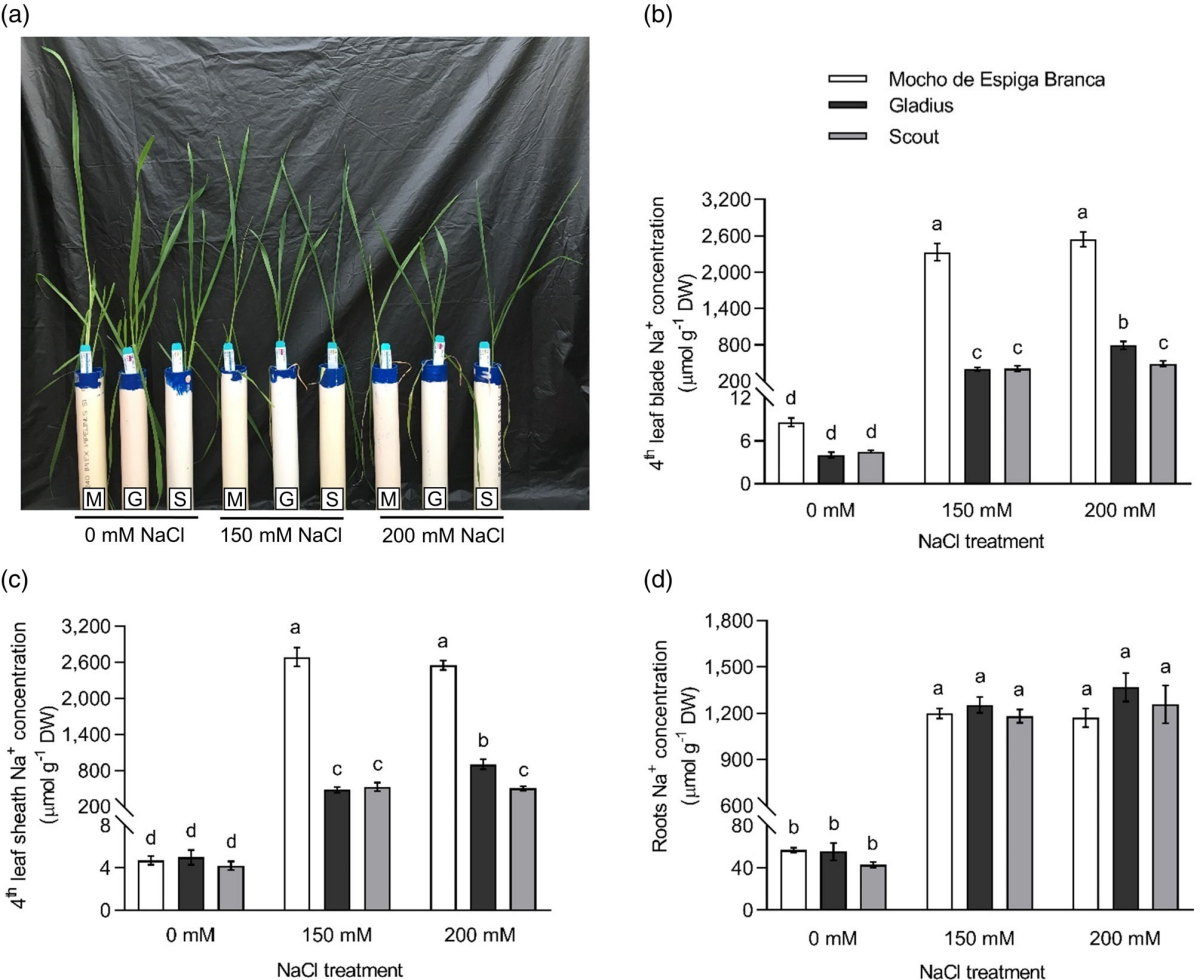
Na^+^ concentration in the fourth leaf blade, sheath, and roots of Mocho de Espiga Branca, Gladius and Scout in hydroponics. (a) Representative image of 6‐week‐old plants from the hydroponic experiment with 0, 150 and 200 mM NaCl treatments applied at the emergence of the fourth leaf. M = Mocho de Espiga Branca, G = Gladius and S = Scout. Na^+^ concentration in (b) fourth leaf blade; (c) fourth leaf sheath and (d) roots determined 21 days after treatments with 0, 150 and 200 mM NaCl. Data presented as means ± SEM (*n* = 14). Bars with different letters indicate significant differences determined by two‐way ANOVA with Tukey's multiple comparison test at *p* ≤ .05 [Colour figure can be viewed at wileyonlinelibrary.com]

In hydroponics, Mocho de Espiga Branca maintained a similar shoot and root biomass to Gladius and Scout at 0, 150 and 200 mM NaCl (Figure 2a and Figure S1a,b), and all three cultivars had comparable salinity tolerance at 150 mM (0.69, 0.64 and 0.60, respectively) and 200 mM NaCl (0.39, 0.49 and 0.39, respectively) (Figure S1c,d). The fourth leaf blade and sheath Na^+^ concentrations were up to sixfold higher in Mocho de Espiga Branca than Gladius and Scout at 150 mM NaCl (Figure 2b,c). At 200 mM NaCl, fourth leaf blade and sheath Na^+^ concentrations in Mocho de Espiga Branca were fivefold greater compared to Gladius and Scout (Figure 2b,c). There was no difference in root Na^+^ concentration at all NaCl treatments (Figure 2d).

All three cultivars had similar K^+^ concentrations in the fourth leaf blade and sheath under 0 mM NaCl (Figure S2a,b). At 150 and 200 mM NaCl, Mocho de Espiga Branca accumulated 70–79% less K^+^ in the blade and 61–67% less K^+^ in the sheath compared to Gladius and Scout (Figure S2a, b). The root K^+^ concentration in Mocho de Espiga Branca was similar to Scout but significantly lower than Gladius under 0 mM NaCl, while no differences were observed between the three cultivars under 150 and 200 mM NaCl (Figure S2c).

The fourth leaf blade and sheath Cl^−^ concentrations in Mocho de Espiga Branca, Gladius and Scout were similar under 0 mM NaCl (Figure S2d,e). Mocho de Espiga Branca accumulated significantly higher Cl^−^ than Gladius and Scout in both tissues under 150 and 200 mM NaCl (Figure S2d,e). There were no significant differences in root Cl^−^ concentrations at 0 mM NaCl (Figure S2f). Mocho de Espiga Branca had significantly higher Cl^−^ than Gladius and Scout at 150 mM NaCl but only significantly higher than Scout at 200 mM NaCl (Figure S2f).

### 3.2 A natural single nucleotide substitution in the *TaHKT1*;*5‐D* gene of Mocho de Espiga Branca alters Na^+^ transport properties of the protein

**FIGURE 3:**
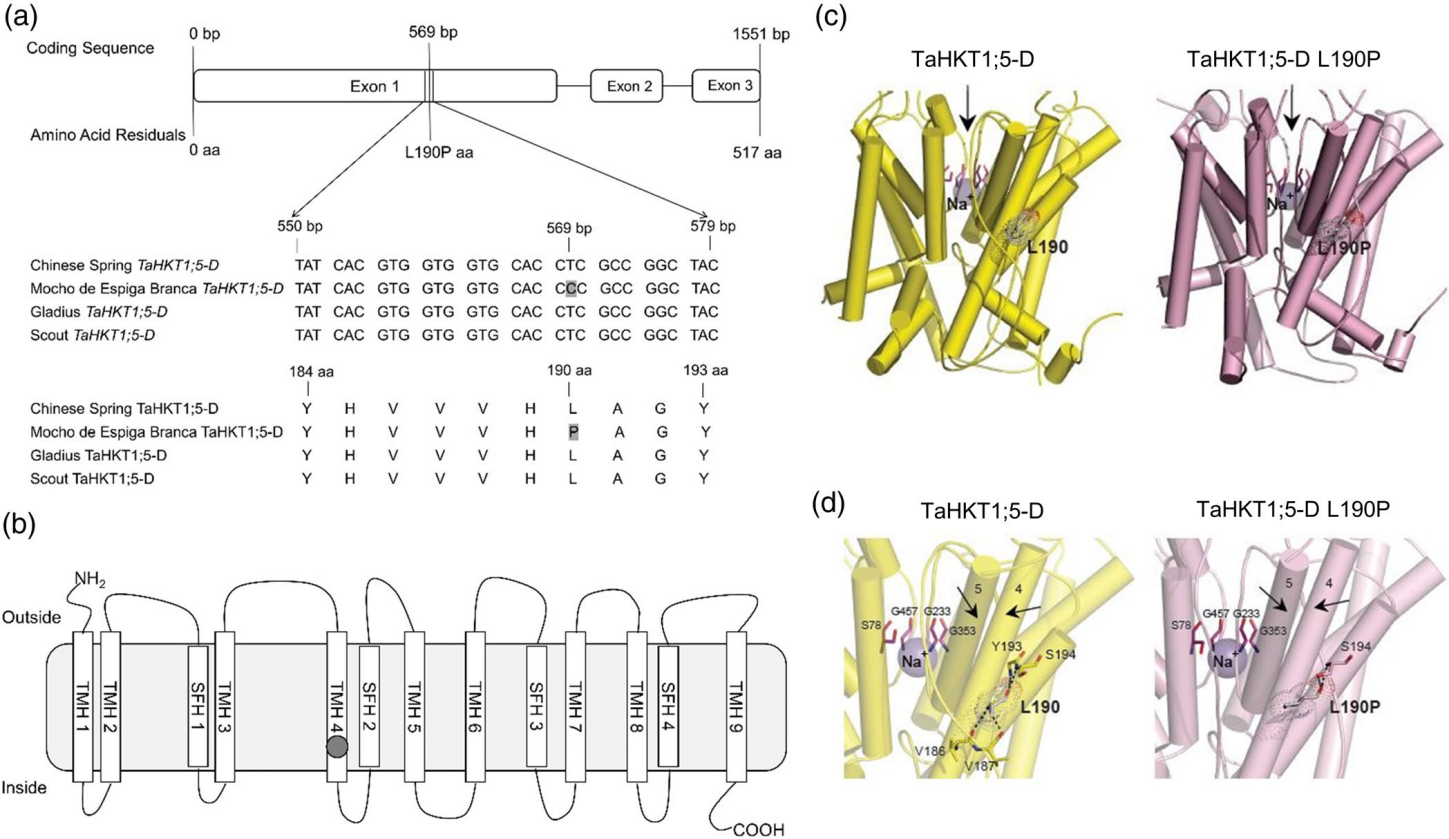
A SNP in Mocho de Espiga Branca *TaHKT1*;*5‐D* results in a L190P amino acid residue variation in the Na^+^ transporter TaHKT1;5‐D. (a) Partial alignment of *TaHKT1*;*5‐D* coding and amino acid sequences in Mocho de Espiga Branca, Gladius and Scout compared to Chinese Spring. (b) Schematic of the TaHKT1;5‐D protein showing the transmembrane α‐helices (TMH 1–9) and selectivity filter α‐helices (SFH 1–4) adapted from Xu et al. (2018); the Na^+^ selectivity filter motif S78‐G233‐G353‐G457 indicated in blank circles and location of the L190P variant indicated in a grey circle. (c) Molecular models of TaHKT1;5‐D (left, yellow) and TaHKT1;5‐D L190P (right, salmon) transport proteins in cartoon representations with cylindrical α‐helices illustrating 3D folds. Constrictions in selectivity filters are bound by four residues (cpk magenta sticks) that contain Na^+^ ions (violet spheres). Black arrows illustrate directional flows of Na^+^ that are likely to enter the permeation trajectory by‐passing selectivity filter constrictions. Variant residues L190 and L190P (cpk sticks and dots, bold types) between wild‐type TaHKT1;5‐D and the L190P mutant are indicated; the dots illustrate volumes of van der Waals radii. (d) Detailed views of α‐helices, which neighbour selectivity filter constriction, containing Na^+^ (violet spheres), located near selectivity filter residues S78, G233, G353, G457 (cpk magenta) for TaHKT1;5‐D (left) and the L190P mutant (right), which are crucial for permeation function. In each protein, polar contacts (cpk sticks and dots) of L190 (TaHKT1;5‐D) and L190P (TaHKT1;5‐D L190P) positioned on α‐helices 4, are indicated by dashed lines (separations between 2.6 Å and 3.2 Å) [Colour figure can be viewed at wileyonlinelibrary.com]

A natural single SNP (T/C) in the coding sequence of *TaHKT1*;*5‐D* was identified at the 569th base pair in Mocho de Espiga Branca, while the sequence of Gladius and Scout was identical to Chinese Spring (Figure 3a). The SNP in Mocho de Espiga Branca resulted in an amino acid residue change from Leucine (L) to Proline (P) at the 190th residue in the Na^+^ transporter protein TaHKT1;5‐D (Figure 3a). This L190P variant residue is predicted to be located on the fourth transmembrane α‐helix in the area of the second glycine residue of the S78‐G233‐G353‐G457 selectivity filter motif (Figure 3b). Expression analysis of the *TaHKT1*;*5‐D* in Mocho de Espiga Branca, Gladius and Scout showed no significant difference in expression (Figure S3a,b). A cleaved amplified polymorphic sequence (CAPS) marker tsl2SALTY‐4D designed to this SNP in *TaHKT1*;*5‐D* was used to genotype 71 diversity lines (Figure S3c and Table S2). Mocho de Espiga Branca carried the C:C allele responsible for the TaHKT1;5‐D L190P variation, while all other lines had the T:T allele as Gladius and Scout (Figure S3c and Table S2).

Three‐dimensional molecular modelling revealed that overall folds of TaHKT1;5‐D and TaHKT1;5‐D L190P were likely to be similar (Figure 3c,d). Detailed analysis of the microenvironments around α‐helix 4 and α‐helix 5 (two black arrows pointing to each other in Figure 3d), revealed that L190 in α‐helix 4 of TaHKT1;5‐D (Figure 3d left) established four polar contacts at separations between 2.6 Å to 3.2 Å with V186, V187, Y193 and S194 neighbouring residues. These extensive polar contacts were not formed in the TaHKT1;5‐D L190P variant (Figure 3d right), which only established one polar contact at the separation at 2.7 Å. In TaHKT1;5‐D, the packing angle between α‐helix 4 (carrying L190P) and the neighbouring α‐helix 5 was 16° sharper than that in TaHKT1;5D L190P (Figure 3d). Sequence conservation patterns, based on 3D models of TaHKT1;5‐D revealed that the P190 position in TaHKT1;5‐D could not be found in databases, meaning that L190 could only be replaced by F, G, L, V, I, M, A, K and T, but not by P. Evaluation of differences of Gibbs free energies of TaHKT1;5‐D revealed that the L190P mutation was energetically highly unfavourable (highly destabilizing), and that the reverse mutation (P190 into L190) restored 70% of this energy loss.

To examine the Na^+^ transport function of the TaHKT1;5‐D L190P variant, *TaHKT1*;*5‐D* cRNA from Mocho de Espiga Branca or Gladius was introduced in *X. laevis* oocytes. When exposed to different concentrations of Na^+^ glutamate (1 and 30 mM Na^+^), the oocytes with *TaHKT1*;*5‐D* from Gladius had a significantly greater Na^+^ elicited inward current than those with *TaHKT1*;*5‐D* from Mocho de Espiga Branca, which had limited current, similar to the H_2_O‐injected oocytes (Figure S4). The TaHKT1;5‐D from Gladius showed a positive reversal potential shift when exposed to 30 mM Na^+^ which was not observed with TaHKT1;5‐D L190P from Mocho de Espiga Branca (Figure S4).

**FIGURE 4:**
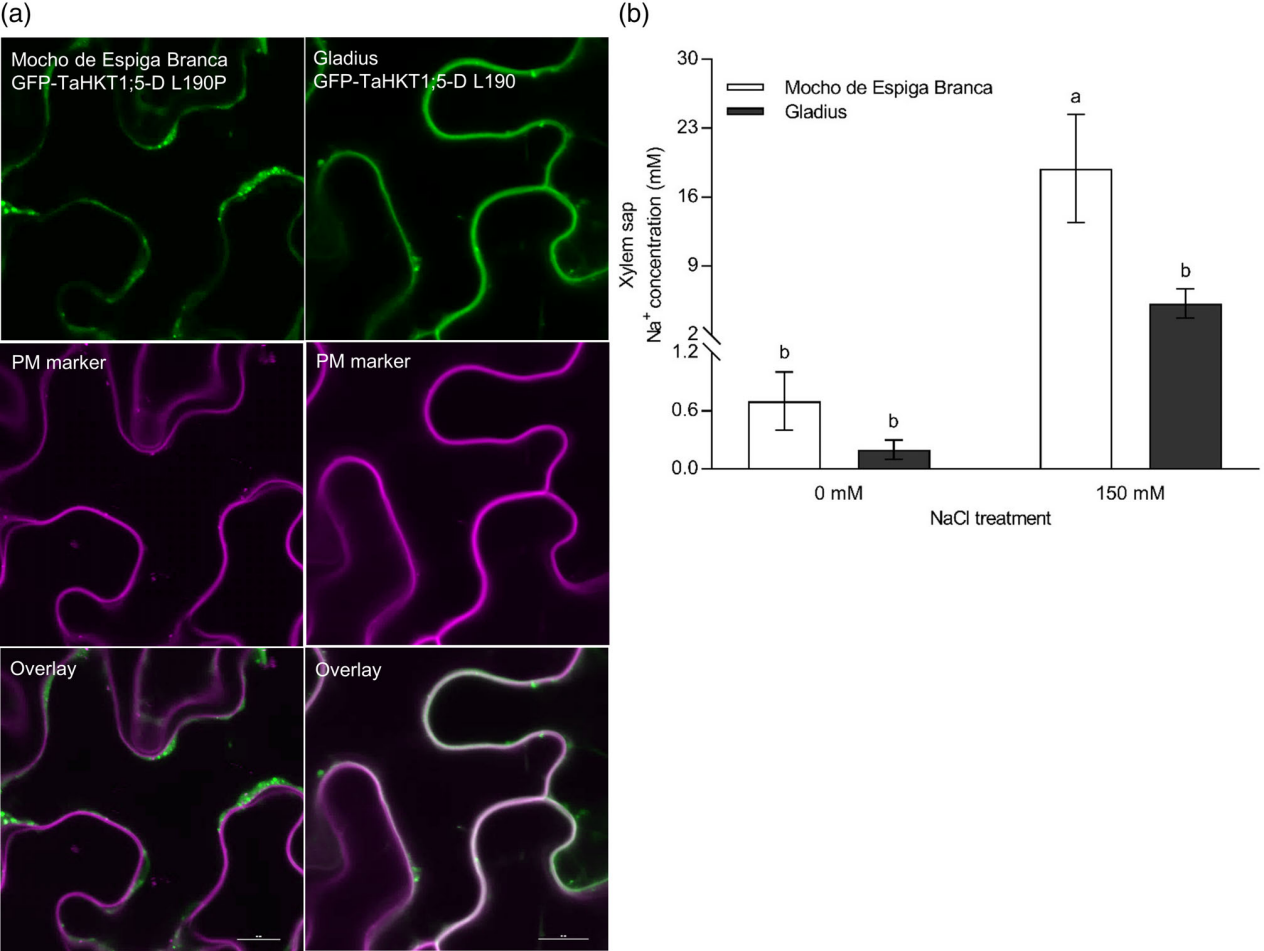
The physiological characterization of L190P variation in TaHKT1;5‐D and the evaluation of the xylem sap Na^+^ concentration in Mocho de Espiga Branca. (a) Transient co‐expression of GFP‐TaHKT1;5‐D variants with a plasma membrane marker in *N. benthamiana* epidermal cells. Leaves were co‐infiltrated with *Agrobacterium tumefaciens* strains harbouring either GFP‐TaHKT1;5‐D L190 (Gladius) or L190P (Mocho de Espiga Branca) and a plasma membrane marker CBL1n‐RFP. GFP signal is shown in green in the left panel while RFP‐signal is shown in magenta in the middle panel. Representative images are shown. Scale bars = 10 μm. (b) The xylem sap Na^+^ concentration of Mocho de Espiga Branca and Gladius under 0 and 150 mM NaCl concentrations. Xylem sap was collected from hydroponically grown plants 21 days after 0 and 150 mM NaCl was applied at the emergence of fourth leaf. Bars with different letters indicate significant differences determined by two‐way ANOVA with Tukey's multiple comparison test at *p* ≤ .05 [Colour figure can be viewed at wileyonlinelibrary.com]

Transient expression of N‐terminally GFP‐tagged TaHKT1;5‐D variants in *Nicotiana benthamiana* leaves revealed differences in GFP‐signal pattern (Figure 4a). The majority of GFP‐TaHKT1;5‐D signal co‐localized with the plasma membrane (PM) marker CBL1n‐RFP, and a minor fraction to mobile subcellular organelles (Figure 4a). GFP‐signal in leaves infiltrated with GFP‐TaHKT1;5‐D L190P, however, localized to internal cell structures, including a faint cytosolic signal and brighter non‐mobile structures (Figure 4a). These data suggest that the TaHKT1;5‐D variant protein could undergo misfolding/unfolding or proteolysis; this may result in a non‐functional protein (as shown by homology modelling) or in an incorrect insertion of the TaHKT1;5‐D L190P protein in a membrane.

The Na^+^ transport properties of the TaHKT1;5‐D L190P variant was further investigated by comparing the xylem sap Na^+^ concentrations of Mocho de Espiga Branca and Gladius grown hydroponically under 0 and 150 mM NaCl. Mocho de Espiga Branca accumulated a 3.5‐fold greater xylem sap Na^+^ than Gladius at 150 mM NaCl, while no significant differences were observed at 0 mM NaCl (Figure 4b). There was no significant difference for xylem sap K^+^ and Cl^−^ concentrations for either cultivar (Figure S5a,b).

**FIGURE 5:**
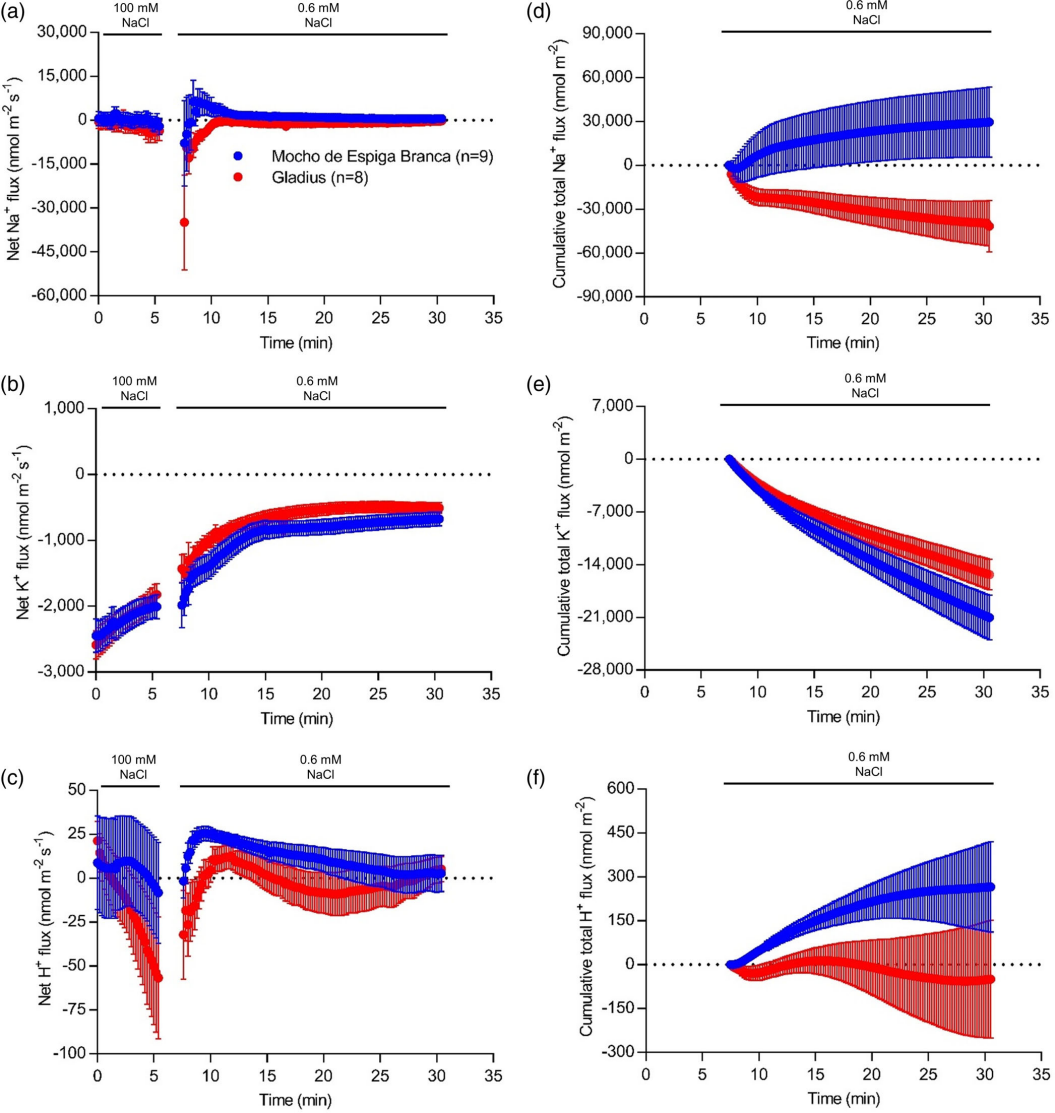
Ion fluxes measured at the root elongation zone after removal from 100 mM NaCl; 6–7–day‐old Mocho de Espiga Branca or Gladius seedlings were treated with 100 mM NaCl for 2 days before removal from the solution, the resultant ion fluxes were measured at the elongation zone (between 200 and 600 μm from the root cap) of the primary root of the plants for 25 min. Net (a) Na^+^; (b) K^+^ and (c) H^+^ fluxes. Cumulative total (d) Na^+^; (e) K^+^ and (f) H^+^ fluxes over 25 min. Data presented as means ± SEM (*n* = 8–9) [Colour figure can be viewed at wileyonlinelibrary.com]

The ability of Mocho de Espiga Branca to retrieve Na^+^ at the root elongation zone was assessed for 25 min by transferring 5‐day‐old seedlings from 100 mM NaCl to a low salt (0.6 mM) basal salt medium (BSM). In the first minute after being exposed to 0.6 mM NaCl, Mocho de Espiga Branca and Gladius had an increase in net Na^+^ efflux up to 10,000 and 35,000 nmol m^−2^ s^−1^, respectively, compared to the BSM with 100 mM NaCl (Figure 5a). However, Mocho de Espiga Branca changed within the first minute to Na^+^ influx, while Gladius maintained Na^+^ efflux for the duration of measurement (Figure 5a). The transient net K^+^ efflux was greater in Mocho de Espiga Branca than Gladius and the efflux rates gradually dropped to below 1,000 nmol m^−2^ s^−1^ in both cultivars (Figure 5b). Mocho de Espiga Branca had a net H^+^ influx for 25 min, while Gladius showed greater H^+^ efflux, or lesser H^+^ influx, than Mocho de Espiga Branca (Figure 5c). Overall, Mocho de Espiga Branca had greater total Na^+^ influx, K^+^ efflux and H^+^ influx compared to Gladius at the root elongation zone (Figure 5d–f).

Upon sudden salt (100 mM) both cultivars had net Na^+^ influx for the first few minutes followed by a gradual net efflux at the maturation zone of the primary root (Figure S6a). Salt stress also induced K^+^ efflux (Figure S6b) and H^+^ efflux (Figure S6c) in both cultivars. However, there was no significant difference in net or total Na^+^, K^+^, and H^+^ fluxes observed between the two cultivars (Figure S6a–f).

## 4 DISCUSSION

A bread wheat landrace, Mocho de Espiga Branca, was identified with significantly higher leaf Na^+^ concentrations and the ability to maintain growth under high salinity. DNA sequencing revealed a naturally occurring SNP in the coding region of the Na^+^ transporter TaHKT1;5‐D, resulting in a L190P amino acid residue change.

Three‐dimensional structural modelling of TaHKT1;5‐D and TaHKT1;5‐D L190P revealed how the L190P substitution impacts protein structure. The lack of the cooperative binding networks around α‐helix 4 of TaHKT1;5‐D L190P within the P190 environment (Figure 3d) is predicted to impose a severe structural rigidity on the 3D folding of this protein. The more obtuse packing angle between α‐helix 4 and α‐helix 5 of TaHKT1;5‐D L190P compared to that in TaHKT1;5‐D, indicated that the proline position affects packing of α‐helices in this specific environment (Figure 3d). Therefore, this α‐helix is unlikely to function properly in the structural context, preventing Na^+^ ion conductance. In TaHKT1;5‐D, a positive correlation was identified between structural characteristics of α‐helix 4 and α‐helix 5 (trends in angles based on α‐helical planes), differences in Gibbs free energies of forward (L190P) and reverse (P190L) mutations and the ability to conduct Na^+^. Unlike TaHKT1;5‐D, the Mocho de Espiga Branca L190P variant was unable to transport Na^+^ following the injection of its cDNA in *X. laevis* oocytes (Figure S4).

At the same time, differences between the sub‐cellular localization of the Mocho de Espiga Branca L190P variant and common TaHKT1;5‐D were identified using transient expression in a plant system. While TaHKT1;5‐D localized on the plasma membrane, the L190P variant exhibited greater localization of GFP signal in internal structures (Figure 4a), suggesting the protein is retained internally and/or is being targeted for degradation. The definitive reason why the TaHKT1;5‐D L190P protein did not transport Na^+^ in *X. laevis* oocytes will need to be established in a subsequent study. However, regardless of whether function is disrupted due to a conformational change, being degraded or mislocalized (or being poorly expressed in oocytes), Mocho de Espiga Branca was still not able to retrieve Na^+^ from the xylem (Figure 4b), explaining why this accession has high leaf blade and leaf sheath Na^+^ (Figure 2b,c).

This work is, to our knowledge, the first that shows a naturally occurring mutation in *TaHKT1*;*5* directly affects both the Na^+^ transport function of the protein and the plant phenotype. Previously, differences in the amino acid sequences between Nipponbare and Pokkali *OsHKT1*;*5* were hypothesized to be responsible for differences in shoot Na^+^ accumulation; however, the transport properties of these proteins were not directly tested (Cotsaftis et al., 2012), and a similar observation was recently made in barley (van Bezouw et al., 2019). Similarly artificially induced mutations in TmHKT1;5‐A, which occurred during cloning of the gene, were shown to disrupt Na^+^ transport properties in *X. laevis*, but this was not linked to a plant phenotype (Xu et al., 2018). A similar natural HKT1;5 variant (L189P) has recently been identified in barley accessions accumulating high grain Na^+^ concentration, which also lacked the ability to transport Na^+^ in *X. laevis* oocytes and was similarly shown to be on internal subcellular structures (Houston et al., 2020).

Other *TaHKT*s may have different expression profiles in Mocho de Espiga Branca compared to Gladius, which may contribute to alternative salt tolerance mechanisms. However, it does not appear that they are involved in retrieval of Na^+^ from the root xylem as the shoot Na^+^ and xylem Na^+^ concentrations were significantly higher in Mocho de Espiga Branca compared to Gladius (Figures 2b,c and 4b). Both Mocho de Espiga Branca and Gladius had similar concentrations of K^+^ in the xylem sap (Figure S5a), however Mocho de Espiga Branca accumulated less K^+^ in the leaf blade and sheath (Figure S2a,b). The greater K^+^ efflux at the root elongation zone in Mocho de Espiga Branca (Figure 5b,e) suggests that the significant reduction in K^+^ in the leaf blade and sheath could be associated with redistribution of K^+^ from shoot to the root in the phloem (Chérel, Lefoulon, Boeglin, & Sentenac, 2013; Dreyer, Gomez‐Porras, & Riedelsberger, 2017) and thereby lowering shoot K^+^ and enabling higher K^+^ root efflux (Figure 5). Root Na^+^ and K^+^ concentrations were similar between the two cultivars (Figure 2d and Figure S2c), because the rate of Na^+^ entry into and K^+^ efflux from, root tissue were similar (Figure S6) for both cultivars during salt stress. In both cultivars, a high concentration of Cl^−^ was transported in the xylem sap (Figure S5b), which accumulated in the leaf sheath to a greater extent than the leaf blade (Figure S2d,e). This is in agreement with previous findings of Cl^−^ partitioning into the leaf sheath in response to salinity and suggests that the leaf sheath may have an important role in Cl^−^ exclusion preventing it from accumulating to toxic concentrations in the leaf blade (Boursier & Läuchli, 1989; Boursier, Lynch, Lauchli, & Epstein, 1987).

**FIGURE 6:**
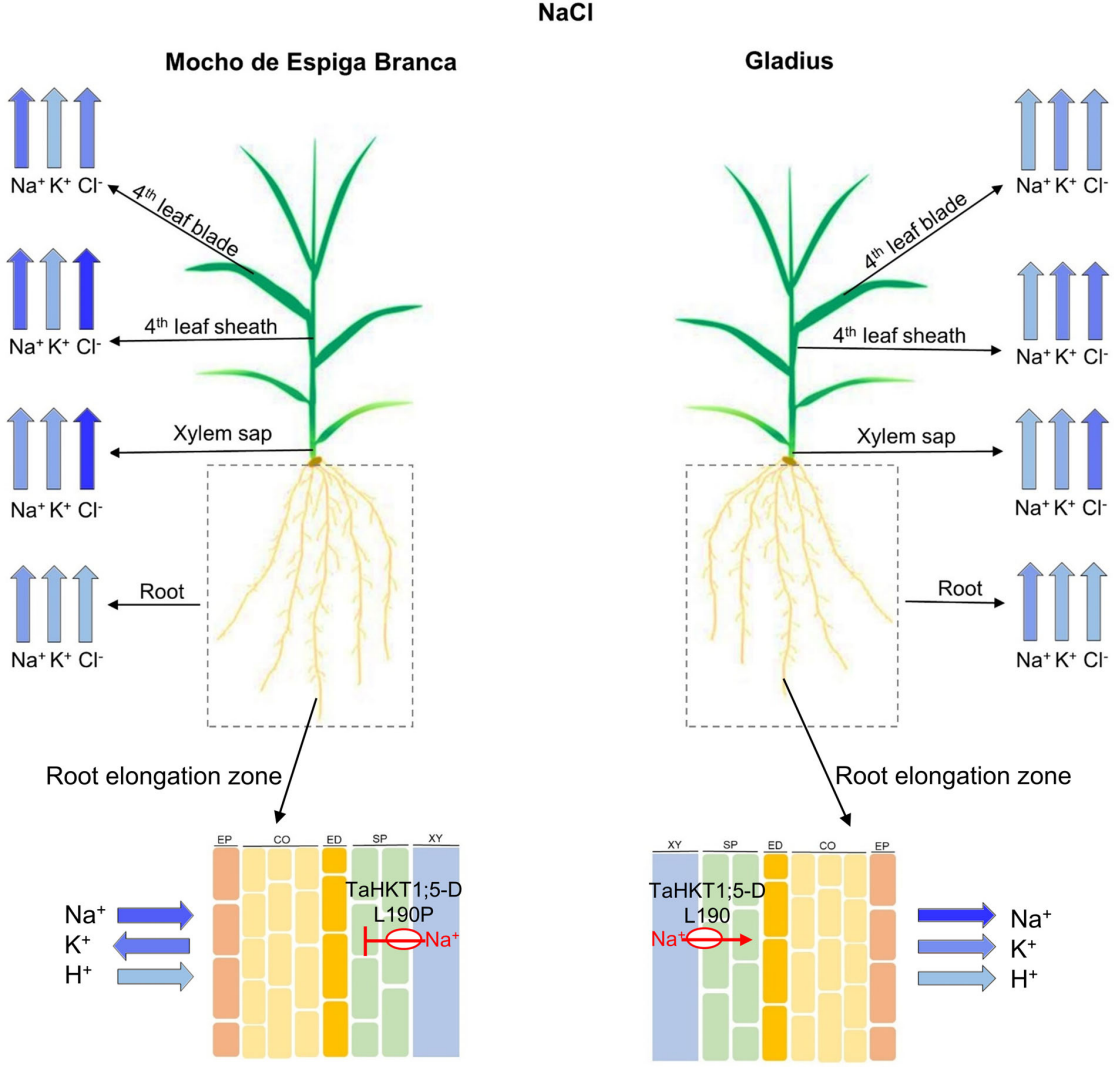
Ion transport model for Mocho de Espiga Branca and Gladius plants under NaCl stress. Wide blue arrows represent the concentration of Na^+^, K^+^, Cl^−^ and H^+^ being transported through or to different tissues and organelles (root elongation zone, root, xylem sap, leaf sheath and leaf blade). Direction of the arrows indicates whether the ion is being taken up to the shoot or is being transported into or out of the root. Colour intensity of the wide blue arrows is proportional to the measured ion concentrations, with a greater intensity representing a higher concentration. EP = epidermis, CO = cortex, ED = endodermis, SP = stellar parenchyma and XY = xylem apoplast. TaHKT1;5‐D L190P in Mocho de Espiga Branca and TaHKT1;5‐D L190 in Gladius are indicated in a red circle with their respective Na^+^ transport feature. The TaHKT1;5‐D L190P variant in Mocho de Espiga Branca leads to reduced retrieval of Na^+^ from the xylem into the roots resulting in a greater influx of Na^+^ in the xylem sap and a higher accumulation of Na^+^ in the leaf blade and sheath compared to Gladius. Mocho de Espiga Branca also has greater Na^+^ influx at the root elongation zone compared to Gladius. There was no difference in root Na^+^ concentration between the two cultivars. The K^+^ concentration was also similar between Mocho de Espiga Branca and Gladius both in the roots and xylem sap; however, Mocho de Espiga Branca had less K^+^ in the leaf blade and sheath compared to Gladius. In both cultivars, a high concentration of Cl^−^ was transported in the xylem sap and accumulated to a high concentration in the leaf sheath compared to leaf blade [Colour figure can be viewed at wileyonlinelibrary.com]

Based on the observed functional defects in Na^+^ transport resulting from the TaHKT1;5‐D L190P variant and the ion analysis findings in this study, we propose a model to compare root‐to‐shoot ion transport between Mocho de Espiga Branca and Gladius (Figure 6). Due to the naturally occurring SNP in *TaHKT1*;*5‐D*, we suggest that Mocho de Espiga Branca has impaired retrieval of Na^+^ from the root xylem which results in a greater influx of Na^+^ in the xylem sap and a higher accumulation in the leaf blade and sheath compared to Gladius (Figure 6).

The results of this study suggest the importance of *TaHKT1*;*5‐D* in the Na^+^ exclusion mechanism of bread wheat to limit the levels of Na^+^ that accumulate. It is evident, however, that *TaHKT1*;*5‐D* does not represent the only mechanism responsible for the salinity tolerance of a whole plant, as Mocho de Espiga Branca maintained similar tolerance to Gladius and Scout despite carrying the non‐functional *TaHKT1*;*5‐D*. It appears that although *TaHKT1*;*5‐D* has a key role in Na^+^ exclusion, salinity tolerance in bread wheat may not necessarily be entirely related to the plants ability to maintain a low shoot Na^+^ concentration. The lack of a relationship between shoot Na^+^ concentration and salinity tolerance in bread wheat has been observed in other studies (Genc et al., 2019; Genc, McDonald, & Tester, 2007). Studies looking solely at Na^+^ exclusion may have been missing other tolerance mechanisms which have been masked by an easily measured phenotype. A number of salinity tolerance mechanisms, such as shoot ion tissue tolerance, may be responsible for Mocho de Espiga Branca's ability to tolerate high shoot Na^+^ concentrations under salinity.

When Na^+^ or Cl^−^ is accumulated to a high concentration in shoot tissue, cells are able to compartmentalize ions in vacuoles to avoid accumulating toxic concentration of Na^+^ and/or Cl^−^ in the cytosol, thus maintain sensitive metabolic processes (Flowers et al., 2015; Munns et al., 2016; Munns & Tester, 2008). In order to balance the osmotic pressure induced by Na^+^ and/or Cl^−^ sequestration in the vacuole, organic solutes such as proline and glycine betaine that are compatible with metabolic activities are required to be synthesized in the cytosol (Munns et al., 2016; Munns & Tester, 2008). Specific signalling pathway mechanisms, such as those for ROS, which have been shown to play a role in regulating vasculature Na^+^ concentrations (Mittler, 2019; Mittler et al., 2011; Suzuki et al., 2012), or Ca^2+^ pathways, which regulate gene expression and protein activities, could also be important for the plant and/or cell's ability to tolerate high concentrations of shoot Na^+^ (Kudla, Batistič, & Hashimoto, 2010; Steinhorst & Kudla, 2019; Thoday‐Kennedy, Jacobs, & Roy, 2015). Therefore, future studies towards improving salinity tolerance of bread wheat should focus on identifying genetics and physiological mechanisms involved in the plant's tolerance to high shoot Na^+^ (tissue tolerance) rather than preferentially focusing on Na^+^ exclusion. This will now be easier, knowing it is possible for wheat to survive high shoot Na^+^ concentrations.

In summary, this study identified a bread wheat landrace, Mocho de Espiga Branca, that maintains shoot growth while accumulating very high leaf Na^+^ concentrations under salinity – a novel bread wheat line with tissue tolerance. A naturally occurring SNP variation in the coding region of *TaHKT1*;*5‐D* of Mocho de Espiga Branca results in the amino acid residue substitution L190P in the Na^+^ transporter TaHKT1;5‐D, and this single substitution appeared to negatively affect the Na^+^ transport function of the protein, which results in high leaf Na^+^ concentrations. Having now identified a high shoot Na^+^ accumulating bread wheat well as the development of the marker tsl2SALTY‐4D, for tracking the SNP variation in TaHKT1;5‐D, we can now identify other bread wheat accessions which have the ability to accumulate high shoot Na^+^ concentrations. Studying variation for sodium tissue tolerance in these high Na^+^ accumulating lines will help accelerate the development of more salinity‐tolerant bread wheat cultivars in the future.

## CONFLICTS OF INTEREST

The authors declare that they have no conflict of interest.

## Supporting information


**Figure S1** Plant biomass and salinity tolerance of plants in relation to their fourth leaf Na^+^ concentration in Mocho de Espiga Branca and two Australian cultivars Gladius and Scout.
**Figure S2.** K^+^ and Cl^−^ concentrations in the fourth leaf blade, sheath, and roots of Mocho de Espiga Branca, Gladius and Scout.
**Figure S3.** Expression of *TaHKT1;5‐D* in the root tissue and CAPS marker tsl2SALTY‐4D genotyping of Mocho de Espiga Branca, Gladius and Scout.
**Figure S4.** Current–voltage (I‐V) curve observed from Mocho de Espiga Branca (blue) or Gladius (red) *TaHKT1;5‐D* cRNA‐injected or H_2_O‐injected (black) *X. laevis* oocytes.
**Figure S5.** Xylem sap K^+^ and Cl^−^ concentration of Mocho de Espiga Branca and Gladius.
**Figure S6.** Ion fluxes measured at root mature zone after a sudden exposure to 100 mM NaCl concentration.
**Table S2.** Genotyping of Mocho de Espiga Branca, Gladius, Scout and 68 bread wheat diversity lines using the CAPS marker tsl2SALTY‐4D.
**Table S3.** Primers used for *TaHKT1;5‐D* coding sequence amplification and sequencing.

**Table S1** **Screening of 75 bread wheat lines for salinity tolerance.** The fourth leaf Na^+^, K^+^ and Cl^−^ concentrations, final point projected shoot area (PSA), and salinity tolerance (PSA under 100 mM NaCl relative to 0 mM additional NaCl added treatment) of the plants are determined 11 days after 100 mM NaCl treatment applied at the emergence of fourth leaf (n = 3–4 except for Gladius and Scout, where n = 12). The plants are ordered from highest to lowest based on their fourth leaf Na^+^ concentration in 100 mM NaCl. One‐way ANOVA was performed to determine the least significant differences (LSD) at *p* ≤ .05.
